# Mutations in Radial Spoke Head Genes and Ultrastructural Cilia Defects in East-European Cohort of Primary Ciliary Dyskinesia Patients

**DOI:** 10.1371/journal.pone.0033667

**Published:** 2012-03-20

**Authors:** Ewa Ziętkiewicz, Zuzanna Bukowy-Bieryłło, Katarzyna Voelkel, Barbara Klimek, Hanna Dmeńska, Andrzej Pogorzelski, Anna Sulikowska-Rowińska, Ewa Rutkiewicz, Michał Witt

**Affiliations:** 1 Department of Molecular and Clinical Genetics, Institute of Human Genetics, Polish Academy of Sciences, Poznan, Poland; 2 International Institute of Molecular and Cell Biology, Warsaw, Poland; 3 Department of Lung Physiology, Children's Memorial Health Institute, Warsaw, Poland; 4 Department of Pneumatology and Cystic Fibrosis, Institute of Tuberculosis and Lung Diseases, Rabka, Poland; 5 Department of Developmental Age Pathomorphology, Medical University of Warsaw, Warsaw, Poland; Universität Heidelberg, Germany

## Abstract

Primary ciliary dyskinesia (PCD) is a rare (1/20,000), multisystem disease with a complex phenotype caused by the impaired motility of cilia/flagella, usually related to ultrastructural defects of these organelles. Mutations in genes encoding radial spoke head (RSPH) proteins, elements of the ciliary ultrastructure, have been recently described. However, the relative involvement of *RSPH* genes in PCD pathogenesis remained unknown, due to a small number of PCD families examined for mutations in these genes. The purpose of this study was to estimate the involvement of *RSPH4A* and *RSPH9* in PCD pathogenesis among East Europeans (West Slavs), and to shed more light on ultrastructural ciliary defects caused by mutations in these genes. The coding sequences of *RSPH4A* and *RSPH9* were screened in PCD patients from 184 families, using single strand conformational polymorphism analysis and sequencing. Two previously described (Q109X; R490X) and two new *RSPH4A* mutations (W356X; IVS3_2–5del), in/around exons 1 and 3, were identified; no mutations were found in *RSPH9*. We estimate that mutations in *RSPH4A*, but not in *RSPH9*, are responsible for 2–3% of cases in the East European PCD population (4% in PCD families without *situs inversus*; 11% in families preselected for microtubular defects). Analysis of the SNP-haplotype background provided insight into the ancestry of repetitively found mutations (Q109X; R490X; IVS3_2–5del), but further studies involving other PCD cohorts are required to elucidate whether these mutations are specific for Slavic people or spread among other European populations. Ultrastructural defects associated with the mutations were analyzed in the transmission electron microscope images; almost half of the ciliary cross-sections examined in patients with *RSPH4A* mutations had the microtubule transposition phenotype (9+0 and 8+1 pattern). While microtubule transposition was a prevalent ultrastructural defect in cilia from patients with *RSPH4A* mutations, similar defects were also observed in PCD patients with mutations in other genes.

## Introduction

Primary ciliary dyskinesia (PCD; MIM #242650) is a rare, multisystem disease with the prevalence of 1/20,000 [1]. Characterized by recurrent respiratory infections, bronchiectasis, male infertility, and randomization of body organ symmetry, PCD is primarily caused by the impaired motility of respiratory cilia, spermatozoid flagella and primary cilia of the embryonic node [2], [3]. Genetically heterogeneous, PCD is usually inherited as an autosomal recessive trait [4]–[7]. To date, PCD-causing mutations have been found in twelve genes, encoding proteins involved in the ciliary ultrastructure (*DNAH11*, *DNAI2*, *DNAL1*, *TXNDC3*, *RSPH9*, *RSPH4A*, *CCDC39*, *CCDC40*) or assembly (*KTU*, *LRRC50*) [8]–[26]; mutations in *RPGR* and *OFD1* have been reported in rare syndromic forms of PCD [27]–[30].

In most PCD cases, dysfunction of cilia or flagella is caused by defects of their ultrastructure. The main part of a cilium, the axoneme, is built on a scaffold of microtubules (MT) projecting from the cell surface. In motile cilia and flagella, nine peripheral MT doublets surround the central pair of MTs (9+2); primary cilia lack the central pair (9+0) [1]. Peripheral doublets in 9+2 cilia are associated with a variety of structures, distributed periodically along the MT length: outer and inner dynein arms producing the force needed for ciliary motility, nexin links connecting the neighboring doublets, and radial spokes providing contact between peripheral doublets and the central pair. Transmission electron microscopy (TEM) reveals aberrations of the axonemal ultrastructure in over 80% of PCD patients [31]. The most commonly reported defects involve absence or shortening of dynein arms. Accordingly, mutations in *DNAI1* and *DNAH5* genes encoding outer dynein arm proteins have been collectively estimated to account for 30–40% of PCD cases [23], [32]. Anomalies of MT arrangement comprise another class of frequently observed defects [33], [34]. Mutations in *CCDC39* and *CCDC40* genes, encoding proteins involved in the formation of dynein regulatory complex, have been reported in a considerable number of PCD patients with defects in MT arrangement [24], [26]. *RSPH4A* and *RSPH9*, both encoding radial spoke head proteins, are other genes reported to be mutated in PCD patients with MT defects [19]. However, the fact that only a small number of preselected families have been examined so far leaves the unsolved question of the overall involvement of *RSPH* genes in PCD pathogenesis. Also, data on ultrastructural cilia defects in patients with *RSPH* defects remain scarce.

Here, we report the results of mutation screening in *RSPH4A* and *RSPH9*, performed in a large group of Polish PCD patients recruited without any preselection. Our data indicate that mutations in *RSPH4A*, but not in *RSPH9*, can be considered a relatively frequent cause of PCD in East-European populations. In addition, new TEM data enrich the existing knowledge of a genotype-phenotype correlation in patients with *RSPH4A* mutations.

## Results

### *RSPH4A* gene

#### Gene sequence analysis

**Figure 1:**
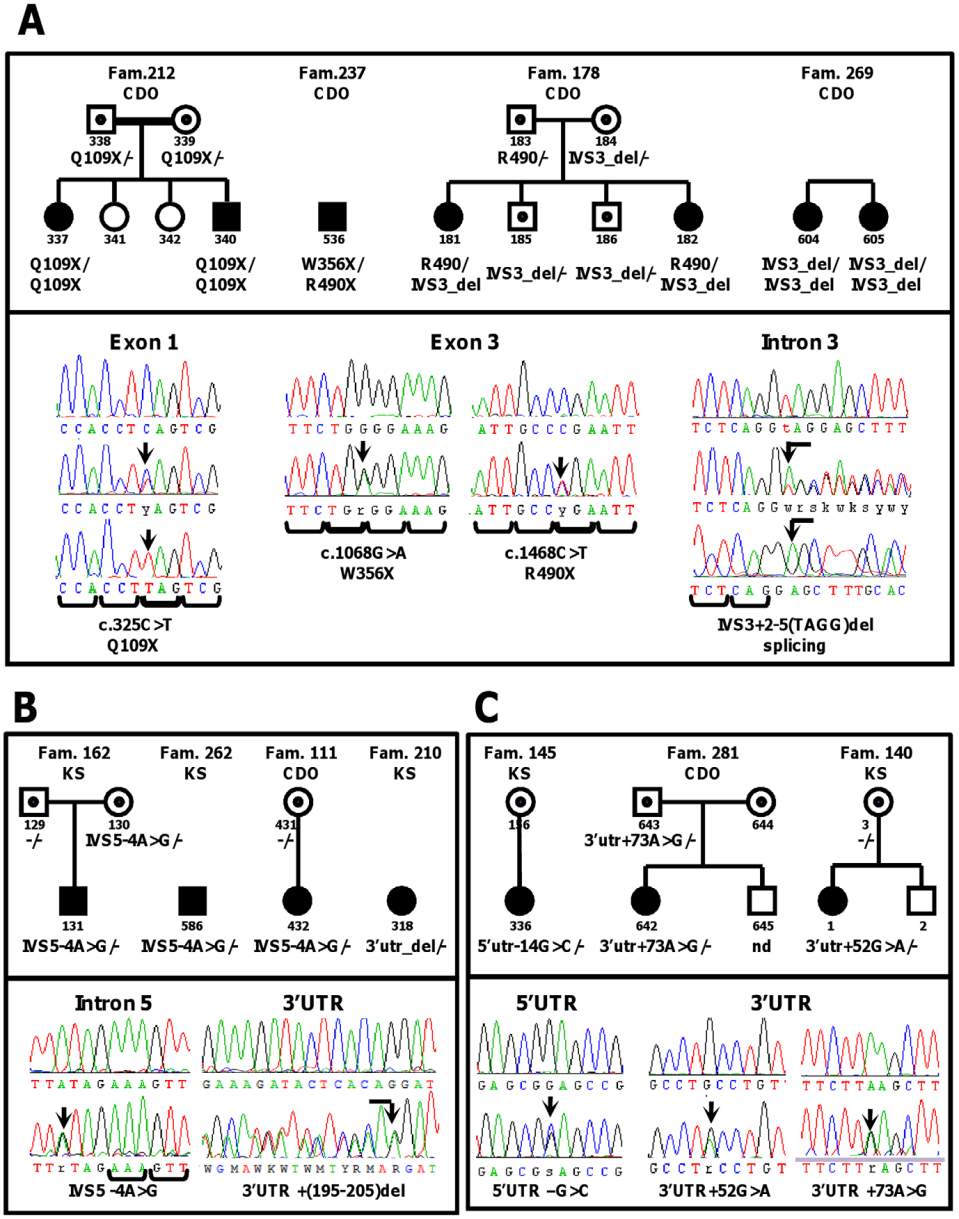
PCD families with new sequence changes identified in *RSPH4A* and *RSPH9* genes. **A** Causative mutations in *RSPH4A*. Segregation of the mutated alleles is consistent with the recessive mode of inheritance. No *situs* inversus was observed in any of the affected members; **B** New SNPs in *RSPH4A*; **C** New SNPs in *RSPH9*. Upper panels – pedigrees of the families; lower panels – sequencing chromatograms.

Fifteen SSCP variants were found upon screening of *RSPH4A* in PCD patients. Nine common SNPs (see next paragraph), previously reported in SNP database, were found also in healthy controls. Analysis of the remaining six variants' occurrence is presented in Fig. 1-A, 1-B and Table 1.

**Table 1:** Details of patients' phenotypes and sequence changes in *RSPH4A*.

					Allele 1			Allele 2		
Family	Patient	s.i.	nNO	Defects	Location	DNA	Effect	Location	DNA	Effect
212	337	no	n.a.	MT	exon1	**325C>T**	**Q109X**	exon1	**325C>T**	**Q109X**
212	340	no	n.a.	MT	exon1	**325C>T**	**Q109X**	exon1	**325C>T**	**Q109X**
237	536	no	75.0	n.a.	exon1	**1068G>A**	**V356X**	exon3	**1468C>T**	**R490X**
178	181	no	44.2	MT	intron3	**IVS3+2–5 (TAGG)del**	**DSS**	intron3	**1468C>T**	**R490X**
269	604	no	125.0	MT	intron3	**IVS3+2–5 (TAGG)del**	**DSS**	intron3	**IVS3+2–5 (TAGG)del**	**DSS**
269	605	no	n.a.	MT	intron3	**IVS3+2–5 (TAGG)del**	**DSS**	intron3	**IVS3+2–5 (TAGG)del**	**DSS**
262	586	yes	n.a.	n.a.	intron5	IVS5 −4A>G	?	-	**-**	**-**
162*	131	yes	n.a.	n.a.	intron 5	IVS5 −4A>G	?	-	**-**	**-**
111	432	no	n.a.	n.a.	intron 5	IVS5 −4A>G	?	-	**-**	**-**
210	318	yes	n.a.	n.a.	3′UTR	3′UTR+195–205(11 bp)del	?	-	**-**	**-**

Causative mutations are shown in bold; the remaining changes (in intron 5 and in 3′UTR) are newly described SNPs. s.i. – *situs inversus*; nNO – nasal NO (parts per billion), average from 3–4 measurements (ozone-chemiluminescence NO analyzer, GE Analytical Instruments); MT – microtubules; n.a. – not analyzed; ? – effect unknown, presumably neutral mutation; DSS – mutation in a conserved donor splice site; an asterisk indicates a family of Slovak origin.

A homozygous c.325C>T transition, introducing a premature stop codon Q109X in exon 1, was found in one family (#212) with two affected siblings. There was reported consanguinity in the family; two unaffected sisters did not carry the mutation. A c.1468C>T transition, resulting in R490X in exon 3, was found in a heterozygous state in two families (#237 and #178). In a solitary proband from family #237, it was accompanied by c.1068G>A, resulting in W356X in exon 3. In two affected sisters from family #178, the R490X was accompanied by a deletion of 4 nucleotides at the intronic positions IVS3+(2–5); the latter mutation was also carried by two unaffected brothers. The IVS3+(2–5)del was also found in a homozygous state in two affected sisters from yet another family (#269), where no information on parental consanguinity had been provided. Although no RNA was available to examine the effect of IVS3+(2–5)del on splicing, we assumed that this variant represented a splice-site mutation, based on a number of observations. First, IVS3+(2–5)del changed the canonical splice site and the first two positions of the deletion were conserved in the interspecies comparison with *RSPH4A* orthologues from 13 Eutherian mammals (Table 2). Second, the deletion was absent from ∼200 unrelated healthy control chromosomes. Finally, *in silico* examination of the effect of IVS3+(2–5)del on the predicted splice sites (Table 3) confirmed that this change abolished the existing donor site, resulting in a frameshift and a premature stop codon.

**Table 2:** Evolutionary conservation of the genomic sequence surrounding newly found sequence changes in the *RSPH4A* gene.

Gene region	Intron3 (+2–5del)	Intron5 (−4A>G)	3′UTR (+195del11 bp)
*Homo sapiens*	**TCAG**GTAGGAGCTTT	TACTT-ATAG**AAA**	AAGATACTCACAGGATTTTCC
*Pan troglodytes*	...............	.....-.......	.....................
*Gorilla gorilla*	---------------	.....-.......	..........T..........
*Pongo abelii*	...............	.....-.......	...........G.........
*Macaca mulatta*	...............	.....-.C.....	..........TG.........
*Callithrix jacchus*	.......A.GA....	.....-.......	.....G....GG.........
*Equus caballus*	.........G.....	A..C.-.C.....	....T.....ACA........
*Sus scrofa*	.........G.....	------------	---------------------
*Bos taurus*	.........G.T...	.....-.C.....	.....G....ACA......T.
*Canis lupus famil.*	.........G.T...	A....T.......	G....G..TGAT..G......
*Mus musculus*	.......T.G.AG.A	CC...-....G..	---------------------
*Rattus norvegicus*	.......ATG..GCC	C....-.C..G..	---------------------
*Oryctolagus cunic.*	.......TAG.....	.T...-....G..	.....................

Mutated sequences are underlined. A point denotes identity, dash – deletion; fragments of the sequences of exon 3 and 6 in *RSPH4A* are shown in bold.

**Table 3:** *In silico* prediction of the effect of IVS3+2–5del in the *RSPH4A* gene.

*RSPH4A* sequence	Splice site	Putative splice site position within the gene sequence	Confidence score	Comparison with the default splice site; effect on splicing
Normal	Donor	TCTCTCTCAĜgtaggagctt	0.63	Default
Normal	Acceptor	gtttccccag^GCAGAAAACG	0.22	Default
Mutated	Donor	GAGGCAGAAĜGTGGGCGAAA	0.55	Alternative in exon; frameshift
Mutated	Donor	aatctatcaĝgtaattaggc	0.41	In intron; frameshift
Mutated	Donor	attgtgccaĝgtgtgtgtgt	0.41	In intron; frameshift
Mutated	Donor	cttggaaaat̂gtatgtagaa	0.32	In intron; frameshift
Mutated	Acceptor	gtttccccag^GCAGAAAACG	0.22	No change

Default splice sites in IVS3 result in the proper protein sequence (donor in phase 2, acceptor in phase 0). All alternative donor sites predicted for the sequence with IVS3+2–5del mutation (located either within exon 3 or within intron 3, all in phase 1) result in a frameshift and a premature stop codon; acceptor site remains unchanged. Intronic sequences in the *RSPH4A* sequence are indicated by lowercase letters. The two most conserved positions of a consensus donor and acceptor splice site are underlined.

The remaining two sequence changes identified in the examined PCD patients were: IVS5 −4A>G and 3′UTR+(195–205)del. No SNPs were reported at the respective gene positions in the human SNP database (build 134). The intronic A>G transition was found in a heterozygous state in three non-related PCD patients. It was absent from ∼400 healthy control chromosomes and, in the interspecies comparison with *RSPH4A* orthologues from 13 mammalian species (Table 2), an A at the IVS5 position −4 was conserved in all these species except *S. scrofa*, for which this segment was not present in the alignment. However, *in silico* analysis did not predict any change in the existing splice sites (data not shown), and no complementing mutation was identified, in spite of sequencing the whole *RSPH4A* coding sequence in the three patients carrying the transition. Two patients carrying this allele had *situs inversus*, and the third patient had no defect of MT organization in TEM; thus, their phenotypes were discordant with that considered characteristic for radial spoke defects (see the next paragraph and Discussion). Based on the above observations, we assumed that IVS5-4A>G did not represent a causative PCD mutation. The deletion of 11 bp in the 3′UTR+(195–205) was found on a single PCD chromosome (with no complementing mutation identified in the patient), and in 5 out of ∼440 examined healthy control chromosomes. We analyzed the possibility that this deletion affected binding site of miRNAs, but no evidence was found of the predicted miRNA in the region of the deletion (data not shown). This segment was also not strongly conserved in the interspecies comparison (Table 2). Based on the above findings, 3′UTR+(195–205)del was considered to represent a neutral polymorphism.

In summary, three sequence changes that resulted in a STOP mutation, and one that affected the conserved donor splice site position, were directly assumed to represent causative PCD mutations. The positioning of body organs in all the patients carrying these mutations was normal, i.e. no *situs inversus* was observed (Table 1).

#### SNP haplotype background of *RSPH4A* mutations

To elucidate, whether a founder effect was responsible for the repeated occurrence of Q109X, R490X and IVS3+(2–5)del mutations, nine-position SNP haplotypes in eight mutation-carrying chromosomes were compared with haplotypes in other PCD and non-PCD chromosomes (Table 4). As expected, two alleles carrying Q109X (c.325C>T) in homozygous siblings #337 and #340 had an identical haplotype background (C-G-G-G-C-g-A-a-t). Another haplotype (C-**A**-G-**A**-C-g-A-a-**c**) was found in three unrelated chromosomes carrying IVS3+(2–5)del, indicating that they shared a common ancestor. The possibility of recurrent mutation events on these three chromosomes was relatively low, since the background haplotype was rare among non-affected chromosomes (∼2%) and relatively recent (separated by three SNP positions from the ancestral haplotype). R490X was found on two independent chromosomes, each on a different background (C-G-G-G-**T**-g-A-a-t and C-G-G-G-C-g-A-a-**c**). This could be explained by either a recombination of the mutation-carrying haplotype or by recurrent mutation events (see Discussion).

**Table 4:** SNP haplotypes in the *RSPH4A* gene.

SNPs/mutations	S1	M1	S2	M2	M3	S3	M4	S4	S5	S6	S7	S8	S9	S10	S11		
**Location**	e1	e1	e2	e3	e3	e3	i3	e4	e4	i4	e5	i5	3′	3′	3′		
**Ancestral allele**	C	C	G	G	C	G	N1	G	C	G	A	A	N2	A	T		
**Haplotypes**																**PCD**	**Non-PCD**
Neutral 1						A										54	42
Neutral 2																3	1
Mutated 1		**T**														2*	-
Neutral 3											C			C		7	8
Neutral 4	G															24	27
Neutral 5	G									A						5	7
Mutated 2	G			**A**												1	-
Neutral 6									T							87	69
Neutral 7									T			C				3	0
Mutated 3					**T**				T							1	-
Mutated 3r					**T**										C	1	-
Neutral 8								A							C	49	41
Neutral 9								A					D2		C	2	4
Neutral 10			A					A							C	4	3
Mutated 4			A				**D**1	A							C	3	-

SNPs: S1: rs13213314; S2: rs41289942; S3: re117169123; S4: rs6927567; S5: rs784133; S6: 41290844; S7: rs9488991; S8: new; S9: new; S10: rs9488993; S11: rs6925922. Mutations: M1: Q109X (rs118204042); M2: W356X (new); M3: R490X (rs118204043); M4: IVS3+(2–5)del (new). Only the derived (non-ancestral) alleles are indicated in the haplotype variants; causative alleles in the “mutated” haplotypes are in bold. Counts of each haplotype in the examined PCD and non-PCD chromosomes are indicated in two rightmost columns; haplotype frequency distribution did not significantly differ between the affected and non-affected chromosomes (Fisher exact test, not shown). The single haplotype 3r, carrying one of two R490X alleles, contains a putative recombination between the mutation-carrying haplotype 3 and the frequent neutral haplotype 8. An asterisk indicates two mutation-carrying chromosomes found in the single consanguineous family. N1 = TAGG in IVS3_2–5; N2 = GATACTCACAG in 3′UTR; D1 = TAGG deletion in intron 3; D2 = GATACTCACAG deletion in 3′UTR; e – exon; i – intron; 3′ – 3′UTR.

#### Effect of *RSPH4A* mutations on the ciliary ultrastructure

**Figure 2:**
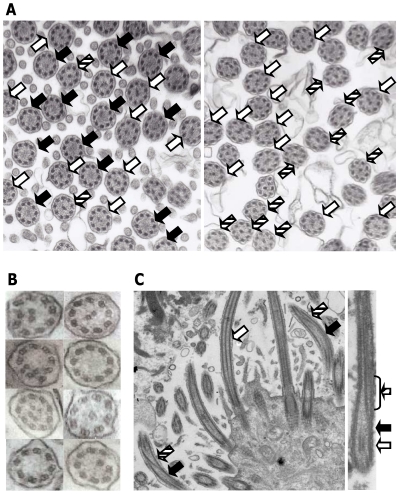
Transmission electron microscope analysis of the bronchial epithelium samples from PCD patients with *RSPH4A* mutations. **A** Ciliary cross sections – magnification 30,000 (patient #181). *Left panel* – a typical picture of proximal ciliary cross sections (cilia are accompanied by numerous microvilli); *right panel* – ciliary cross sections taken at the distance from the cell membrane (no microvilli present); **B** Examples of MT defects in patients #181, #337 and #340 (a blown-up view). **C** Longitudinal sections of axonemes (patient #337); *left panel*: magnification 16,000; *right panel* – a blown up view of a single cilium. White, black and hashed arrows indicate 9+2, 9+0 and 8+1 MT arrangement, respectively.

TEM data were available for three of the four families with *RSPH4A* mutations. In all these cases, analyses revealed defects of MT organization, albeit a significant proportion of ciliary cross-sections with a normal 9+2 pattern were also present (Fig. 2-A; Table 5). An absence of the central pair (9+0) was the most frequently found MT defect. In many cilia, the lack of the central pair was associated with various stages of a displacement of one of peripheral doublets (8+1). Jointly, 9+0 and 8+1 patterns represented 38–54% of all cross-sections examined. Other MT abnormalities, including 9+1, 9+4, 9+3, 8+0, 8+2, 7+1 were rare (none was found in more than 6% of the cross-sections examined). Cilia with a 9+1 pattern were seen only in patient #181 (2%), while cilia with supranumeral central MTs (9+4 and 9+3) were observed more frequently in the siblings #337 and #340 than in #181 (8% vs 3%). The differences in the proportion of 9+4 and 9+1 cilia could be related to the differential effect of underlying mutations (#337/#340 were Q109X/Q109X homozygotes and #181 was a compound heterozygote W356X/R490X); the increased proportion of cilia with rare, non-specific patterns in #337 and #340 most probably represented acquired abnormalities. Ciliary disorientation, with a discordant alignment of the central pair in neighboring cilia, was observed in all patients, in all the fields examined. Radial spokes, nexin links and inner dynein arms were not distinguishable, even in cross sections with the normal MT pattern; this could be a part of the *RSPH4A* defect-related picture, but could also reflect an inadequate quality of specimens. Outer dynein arms appeared normal, both in 9+2 axonemes and in those with defective MT arrangements (Fig. 2-B). The latter observation confirmed that the frequently observed 9+0 pattern reflected the loss of the central pair rather than a fortuitous localization of the cross-section plane at the transition zone of the axoneme. In the specimen from patient #181, where TEM pictures of 14 different fields were available for the analysis, two classes of cross-sections were observed – proximal (as indicated by the large number of microvilli), and more distal, without microvilli (Fig. 2-A). The proportion of 9+0 patterns was much higher among the proximal cross sections, while in the distal part of the axoneme, 9+0 was largely replaced by 8+1, with the transposed peripheral doublets positioned either asymmetrically or in the center of the axoneme (Table 5). Longitudinal sections of a few cilia from patient #337 confirmed the presence of a classical ciliary transposition defect, but also showed that some of the cilia had the central pair retained throughout the whole length of an axoneme (Fig. 2-C).

**Table 5:** MT defects in ciliary cross-sections from patients with *RSPH4A* mutations.

Cross-section details	Patient #181		Patient #337	Patient #604
Microvilli	Numerous	Absent	Few	Nd
Cross-section plane	Proximal	Distal	Ambiguous	Nd
Number of fields examined	7	7	4	1
**9+2 (normal MT pattern)**	**62 (35%)**	**78 (47%)**	**37 (37%)**	**2**
**9+0**	**83 (47%)**	**28 (15%)**	**26 (27%)**	**5**
8+1 C	6	38	5	0
8+1 E	7	12	6	0
8+1 P	3	4	1	0
**All 8+1**	**16 (9%)**	**54 (30%)**	**12 (12%)**	**0**
9+1	6	6	0	0
9+4	4	2	6	0
9+3	2	3	1	0
7+1	1	2	0	0
8+2	1	2	2	2
8+0	1	1	1	0
Various	1	5	14	0
**All other than 9+2, 9+0 and 8+1**	**16 (9%)**	**21 (8%)**	**24 (22%)**	**2**
**Total count of examined cilia**	**177**	**182**	**98**	**7**

Letters C, E and P next to the 8+1 MT pattern denote position of a transposed doublet in the axoneme: central, eccentric and at the perimeter, respectively. Nd: cross-section plane and the presence of microvilli not determined, due to the low number of ciliary cross-sections.

### *RSPH9* gene

SSCP screening of the entire coding region of *RSPH9*, performed in PCD patients, revealed four sequence variants. One, in exon 5, was a known SNP (rs16896629). Three other changes (Fig. 1-C) have never been described before. A heterozygous −14G>C transversion in the 5′UTR was found in a single PCD patient, with no other accompanying mutation identified in the entire coding region. This variant was not found among 186 healthy control chromosomes. Two heterozygous transitions, 52A>G and 73G>A, were found in the 3′UTR, each in one of two unrelated patients; no accompanying mutations were identified. Both transitions were absent from ∼210 chromosomes from unrelated Polish non-PCD individuals. Interrogation of the human SNP database (build 134) did not indicate SNPs at the respective gene positions.

**Figure 3:**
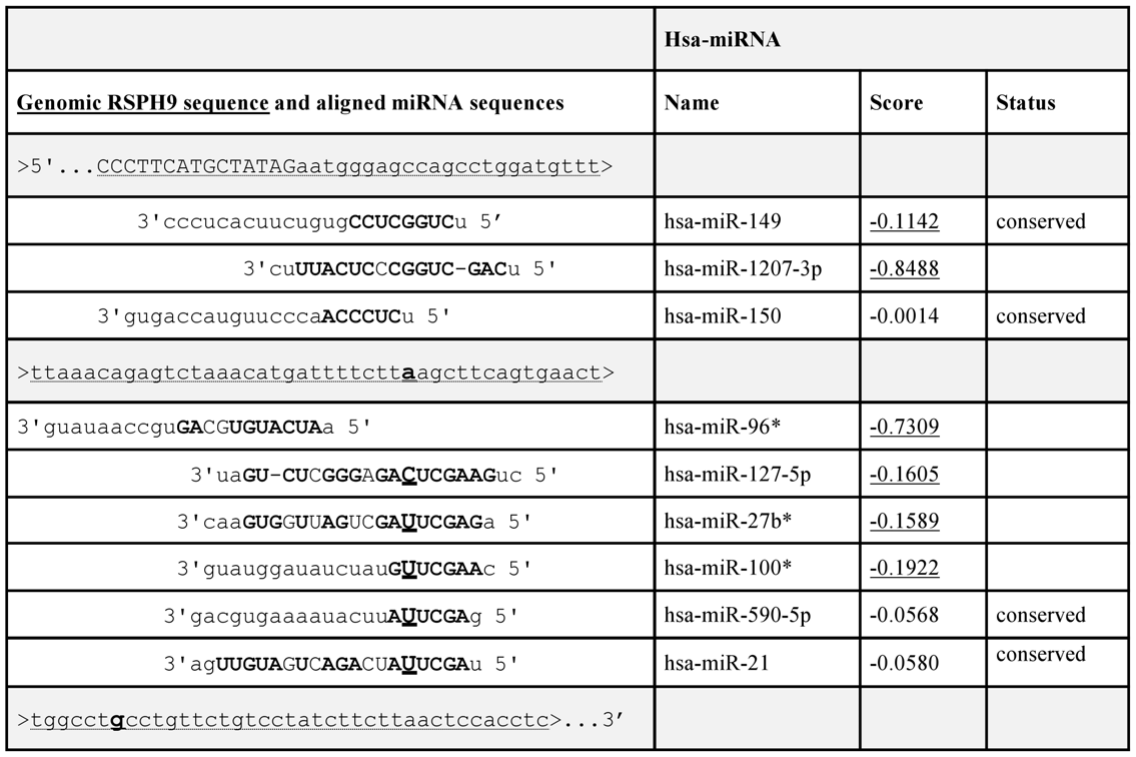
Localization of the sequences recognized by *Homo sapiens* micro RNAs (Hsa miRNAs) within the 3′UTR of *RSPH9* sequence. The genomic sequence (last 15 bp of exon 6 and first 105 bp of 3′UTR) shown in the left column is underlined with the dotted line, with the coding sequence and UTR indicated by upper and lower case, respectively; positions +52A and +73G, mutated in some samples, are indicated (bold and heavy underline). MiRNA sequences identified through in silico search are shown below, aligned with the genomic sequence, with the complementary bases shown in uppercase. MirSVR scores (support vector regression algorithm for the prediction of the miRanda-predicted microRNA target sites; [38]) are indicated next to each miRNA name; high scores are underlined. The A at position +52 in the 3′UTR sequence is complementary to U in four miRNAs; the A>G transition at +52 would increase complementarity between 3′UTR and the miR-127-5p. No miRNAs complementary to the sequence encompassing +73 in the 3′UTR were found.

The biological meaning of the three newly found mutations is difficult to establish without analyzing cDNA, which was not available. Comparison with the *RSPH9* orthologues from 13 Eutherian mammals indicated 100% conservation of A at position 52 of the 3′UTR; 73G in the 3′UTR and −14G in the 5′UTR were less conserved (Table 6). The possibility that the 3′UTR mutations affected binding site of miRNAs was examined using the MIRANDA online tool. The 52A was found to be a part of the sequence recognized by several miRNAs (Fig. 3). The A>G transition at this position would increase the complementarity score for miR-127-5p (A∶C>G∶C), at the same time retaining the complementarity of four other miRs (A∶U>G∶U in 27b*, 100*, 590-5p and 2). Verification of the possibility that 52A>G transition affected the level of *RSPH9* expression would require extensive experimental analyses; this issue was not pursued further, given that a heterozygous 52A>G was found in only one patient with no other changes in *RSPH9*. In summary, we conservatively assumed that none of the newly identified alleles in *RSPH9* represented causative PCD mutations.

**Table 6:** Evolutionary conservation of the genomic sequence surrounding newly found sequence changes in the *RSPH9* gene.

Gene region	5′UTR (−20G>C)	3′UTR (+52A>G and +73G>A)
*Homo sapiens*	TGAGCGGAGCCGCT	TTCTTAAGCTTCAGTGAACTTGGCCTGCCTGT
*Pan troglodytes*	..............	................................
*Gorilla gorilla*	..............	................................
*Pongo abelii*	.....A........	................................
*Macaca mulatta*	.....A...A....	.........................C......
*Callithrix jacchus*	.....A.......A	...........................GT...
*Equus caballus*	--------------	.......AT.........T....T...TT---
*Canis lupus famil.*	--------------	...A...A.AG.T.....T...CT...TT...
*Sus scrofa*	.......G.AG...	...A...AG...G.C.....G..T.C.TT---
*Bos taurus*	.....A....T..C	...A...AG-..G.....TC...T.G.T....
*Mus musculus*	G...GA..A.G...	...A...AG....ACT-GT.G.....TTT...
*Rattus norvegicus*	G...G...A.G...	...A...AG....ACC-.T.G..T....T...
*Oryctolagus cunic.*	.....T.......G	..TA...ATGGT....T....CC-..C.T...

Mutated sequences are underlined. A point denotes identity, dash – deletion.

## Discussion

Our study represents the first large-scale screening of radial spoke head protein genes performed in PCD families, in our case, ones of East-European (Slavic) origin. The earlier report on mutations in *RSPH4A* and *RSPH9* [19] has been based on a much smaller study group of seven PCD families preselected for the presence of MT defects, and included only a single European family.

We did not detect any causative *RSPH9* mutation among the examined PCD patients. The absence of c.801-803delGAA (K268del), reported earlier in Bedouins from United Arab Emirates and Israel [19], confirms the population-specific character of this mutation. We postulate that the involvement of *RSPH9* in PCD pathogenesis among Europeans, or at least in Slavic populations, is negligible.

On the contrary, our data proved that mutations in *RSPH4A* are responsible for a significant proportion of PCD cases among the Slavic people. Although c.504C>T (Q154X), reported earlier in four Pakistani families [19], was not found (suggesting its Pakistani-specific origin), four other disease-causing *RSPH4A* mutations were identified in four Polish families. Importantly for diagnostic purposes, all causative mutations detected up to date clustered in or around exons 1 and 3.

Two of these mutations (Q109X and R490X), previously reported in a compound heterozygote from a single North UK family, were detected in two Polish chromosomes each. Q109X was found in homozygous siblings from a single family; both Q109X alleles had the same SNP haplotype background, consistent with the declared parental consanguinity. Lack of SNP haplotype data in the previous report [19] prevented us from determining whether Q109X mutations found in Polish and UK families were identical by descent. For the same reason, we could not determine a shared or independent ancestry of Polish and UK chromosomes with R490X. In the Polish cohort, R490X was found on two different backgrounds. Since the mutation results from the c.1468C>T transition at a fast-mutating CpG dinucleotide [39], the possibility of its redundant origin is high. On the other hand, the C-G-G-G-C-g-A-a-**c** background was not found in any of the examined non-PCD chromosomes, but could be easily explained by a recombination of R490X-carrying C-G-G-G-**T**-g-A-a-t with another frequent haplotype (Table 4), suggesting that R490X on two different backgrounds could in fact be identical by descent. Of the two newly discovered mutations, W356X was found on a single chromosome, while IVS3_2–5del was repeated on three independent chromosomes. All chromosomes carrying IVS3_2–5del shared the same haplotype (C-**A**-G-**A**-C-g-A-a-**c**) that was rare among non-affected chromosomes and separated by three SNP positions from the ancestral haplotype, which indicates that it is evolutionary young [40]. IVS_2–5del appears therefore to be a relatively recent mutation; further studies involving other PCD cohorts are required to elucidate whether this mutation is restricted to Polish population or spread among other European groups.

TEM analysis of ciliary cross-sections from bronchial epithelium of patients with *RSPH4A* mutations revealed that a ciliary MT transposition, with the loss of the central pair (9+0 pattern), accompanied by a translocation of one of the peripheral doublets (8+1 pattern), was the predominant defect of the ciliary ultrastructure. Frequency of cilia with other abnormalities (each representing less than 4% of the cross sections) was not different from that expected in specimens from healthy individuals [41]. The previously reported estimates of the overall proportion of cilia with central MT defects have ranged from ∼14% in patients with transposition defects [33] to 12.5–17% in patients with central pair agenesis [42]. In our study, the 9+0 pattern was seen in 15–47%, and 8+1 in 9–30% of the examined ciliary cross-sections. The proportion of 9+0 and 8+1 cilia depended on the plane of a cross-section (9+0 prevailed in the proximal, and 8+1 in the distal sections), confirming the previous observation that the loss of the central pair in a major proportion of cilia represents a proximal picture of a ciliary transposition defect [43].

Mutations affecting the RSPH4A protein have been described by Castleman et al. [19] as being associated with a classic transposition defect indicative of “a complete central pair loss”. This description is incorrect, since neither a classic ciliary transposition with one of the peripheral doublets transposed to the center, nor even a central microtubular agenesis [42] are associated with a “complete” loss of the central pair [33], [42]. In a transposition defect, the central pair in cilia has been reported to extend only a short distance [44]. In our study, a significant proportion of cross sections with a normal 9+2 pattern (35–37%) was observed not only in the proximal but also in the distal region, indicating that the lack of a functional RSPH4A protein and the resulting defect of radial spoke heads affected MT arrangement only in some of the axonemes. This could suggest that not only the translocation of a peripheral doublet to the center of the axoneme (8+1) [43], but also the lack of the central pair (9+0) could be considered a secondary effect.

Similar to Castleman et al. [19], we found *RSPH4A* mutations only in PCD patients without *situs inversus*. This is consistent with the hypothesis that defects in radial spoke head proteins do not affect functioning of 9+0 nodal cilia and thus do not impair development of body organ symmetry.

The total number of *RSPH4A* mutations identified in our study indicates that this gene is involved in PCD pathogenesis in 2.2% of Polish PCD families, or in ∼4% considering only families without *situs inversus* (Table 7). It is important to emphasize that these estimations are based on the cohort recruited without any preselection with respect to ultrastructural defects. The frequency of *RSPH4A* mutations in Polish PCD population preselected for defects of ultrastructure would be much higher. To assess the effect of such preselection in the calculation of *RSPH4A* involvement, we reexamined descriptions of diagnostic TEM data available for Polish probands (data not shown). In most cases, an absence of dynein arms (outer, outer/inner or inner) was reported in the majority of cross sections, while MT arrangement defects were observed only in a small proportion (less than 10%) of cilia. Since heterogeneous MT defects have been demonstrated in acquired (secondary) ciliary dyskinesia [45], and occasionally even in healthy individuals [41], we did not consider such low frequency MT defects to represent a primary effect of any specific mutation. However, in 24 probands, MT defects were observed in 13–60% of the scored cilia. Mutations in *RSPH4A* were identified in three of these 24 patients, suggesting that mutations in this gene may be involved in over 12% of PCD families with predominant MT defects. The remaining 21 cases with prevalent MT defects could have included patients with mutations in other genes, e.g. *CCDC39* or *CCDC40* recently demonstrated to display characteristic MT arrangement defects [24], [26]. Interestingly, two patients from this subgroup had mutations in *DNAI1* [23], and three had mutations in *DNAH5* (our unpublished data), both genes primarily associated with defects in outer dynein arms. Earlier reports have indicated that in patients with dynein arms defects, only ∼5% of cilia display MT defects [33], [45]; our data show that mutations in dynein arms proteins may be associated with much more prevalent MT defects. They also indicate that, while predominant MT defects may be considered indicative of dysfunctional RSPH4A, they are not specific for mutations in this protein.

**Table 7:** Occurrence of *RSPH4A* mutations among examined European PCD families.

	[19]		This study		All	
Disease	KS	CDO	KS	CDO	KS	CDO
**Cohort size (number of European families)**	0	1	85	99	**85**	**100**
Number of families with mutations detected		1	0	4	**0**	**5**
Number of chromosomes with 325C>T (Q109X)		1	0	2	**0**	**3**
Number of chromosomes with 1068G>A (W356X)		0	0	1	**0**	**1**
Number of chromosomes with 1468C>T (R490X)		1	0	2	**0**	**3**
Number of chromosomes with IVS3+2–5del (splicing)		0	0	3	**0**	**3**
**Total number of chromosomes with ** ***RSPH4A*** ** mutation**					**0**	**10**

The 460C>T (Q154X) mutation (rs118204041), found only in four consanguineous Pakistani families [19], is not included in the Table.

## Materials and Methods

### Ethics Statement

An informed consent was obtained from all the participating individuals or their parents; the research protocol was approved by the Ethics Committee of the Medical University in Poznan.

### Patients

The study group comprised the previously described cohort of 157 PCD families, including 151 of Polish and 6 of Slovak origin (Slovakians are very closely related to Poles), and additional 27 Polish families recruited [23]. In the combined cohort (213 affected), 85 families were classified as Kartagener syndrome, KS (at least one affected member displayed *situs inversus*). The families were recruited without a preselection for ultrastructural defects, although TEM data were available for ∼50% of the probands. At least one of the three criteria listed below had to be fulfilled to include a patient in the PCD cohort: i) typical clinical symptoms (recurrent upper respiratory tract infections, recurrent pneumonia, chronic bronchitis, bronchiectasis, sinusitis and otitis media, and reduced mucociliary clearance as shown by a negative result of a saccharine test) associated with *situs inversus* (85 families); (ii) typical clinical manifestation without *situs inversus*, but with the presence of a defect in the ciliary ultrastructure (41 families); (iii) typical clinical symptoms without *situs inversus*, and the absence of ciliary motility as seen in the light microscope (58 families). Cystic fibrosis was excluded on the basis of a clinical picture and an absence of most frequent *CFTR* mutations [35]. Parental consanguinity was reported in one family; otherwise, consanguinity was neither reported nor formally excluded. The control group consisted of ∼100 non-PCD Polish individuals, and of parental chromosomes that were not transmitted to affected offspring in PCD families

Search for mutations was not executed in 28 families where two mutated alleles have been previously identified in other PCD-related genes [23; our unpublished data]. In *RSPH4A*, the search was discontinued in 16 families, where the segregation of SNP haplotype was inconsistent with that of the disease. However, all 184 PCD families were included in calculating the overall *RSPH4A* involvement in PCD pathogenesis.

### PCR amplification and SSCP/heteroduplex analysis

Genomic DNA was isolated from peripheral blood lymphocytes using a standard salting-out extraction. Primer pairs were designed to amplify all exons (six in both *RSPH9* and *RSPH4A*) and splice junctions, as well as the 5′ and 3′ untranslated (UTR) regions. The length of each amplicon was <300 bp; some exons were analyzed in 2–4 overlapping parts. PCR-amplified segments were denatured and separated in native polyacrylamide gels [23]. Primer sequences, PCR conditions and detailed SSCP conditions are available from the authors upon request.

### Sequence analysis

Nucleotide changes underlying the detected SSCP migration variants were resolved by direct sequencing of PCR products [23]. The reference genomic sequences for *RSPH4A* (previously *RSH3L*) were ENSG00000111834 (www.ensembl.org); exon boundaries were of the 717 amino acid RSPH4A-02 transcript ENST00000229554. The corresponding reference sequences for *RSPH9* were ENSG00000172426; exon boundaries were of the 306 amino acid RSPH9-02 transcript ENST00000372163. The effect of mutations on the predicted splice sites was examined *in silico*, using NetGene2 online software (http://cbs.dtu.dk/services/NetGene2 [36], [37]). The possible effect of 5′UTR mutations on miRNA-binding sites was examined using MIRANDA online tool (http://www.microrna.org/ microrna [38]). Segregation of *RSPH4A* haplotype composed of nine known intragene SNPs was examined in 153 families; converting genotype data into haplotypes was done as previously described [23].

### Transmission electron microscopy (TEM)

Respiratory epithelial cells from bronchial biopsies were obtained during routine diagnostic procedures. The cells were fixed in 2.5% glutaraldehyde in 0.1 M sodium cacodylate buffer at 4°C, post-fixed in 1% osmium tetroxide, dehydrated and embedded in a mixture of propylene oxide and epoxy resin. Ultramicrotome sections were stained with Reynold's lead citrate. Observations were performed at 16–30,000-fold magnification (Philips CM10).
